# Reply to LTE: Does caffeine truly raise muscle carnitine in humans?

**DOI:** 10.14814/phy2.15796

**Published:** 2023-08-31

**Authors:** Benjamin T. Wall, Francis B. Stephens

**Affiliations:** ^1^ Department of Public Health and Sport Science University of Exeter Exeter UK

We recently published an article in Physiological Reports (Wall et al., 2023) where we hypothesized that caffeine ingestion would stimulate Na^+^‐dependent muscle carnitine uptake, which would be evident from an increase in plasma carnitine clearance. Healthy male and female participants ingested a large bolus of 6‐mg/kg body mass (bm) caffeine followed by three smaller 1 mg/kg bm boluses after 2, 3, and 4 h. This approach resulted in an immediate and considerable rise in plasma caffeine concentration, which was sustained in a relatively steady‐state manner throughout the 5‐h experiment. In parallel, we created steady‐state and supraphysiological hypercarnitinemia via the intravenous infusion of L‐carnitine for 5 h. This combination of high circulating levels of caffeine and carnitine resulted in increased plasma carnitine clearance (i.e., 10%–15% lower plasma carnitine concentration during steady‐state) for the final 2 h of experimentation compared to L‐carnitine infusion and placebo ingestion. While we did not measure muscle carnitine content, we have previously demonstrated that hypercarnitinemia combined with hyperinsulinemia (achieved via intravenous infusion of insulin for 6 h) also increased plasma carnitine clearance by ∼15% and resulted in an increase in skeletal muscle total carnitine content of ∼15% (Stephens et al., 2006).

Constantin‐Teodosiu (2023) has written an LTE to Physiological Reports questioning whether we can truly make assumptions of caffeine stimulating carnitine uptake and accumulation in skeletal muscle. The author gives three reasons to support his argument, which as we explain below, are largely based on incorrect interpretation of our methodology and calculations.

Firstly, Constantin‐Teodosiu suggests that the slopes of the rise in plasma carnitine concentration over the first 30 min after L‐carnitine administration were identical between caffeine and placebo and, given the low bioavailability of oral L‐carnitine, no muscle carnitine accretion could have occurred. We would argue that the lines are visibly not identical (Wall et al., 2023) but, more importantly, we are puzzled by why the author thinks we gave an oral dose of L‐carnitine, as this is not mentioned anywhere in the publication. Moreover, the author has misunderstood the basis of calculations of bioavailability, which classically should compare the plasma carnitine concentration following oral vs. intravenous boluses of a given dose, and measure plasma carnitine concentration over multiple timepoints until it returns to baseline, with much greater resolution than a single 30‐min snapshot. Our design of a bolus intravenous L‐carnitine prime immediately followed by a steady‐state infusion, with no oral L‐carnitine ingestion, was clearly not designed to investigate rates of rise or bioavailability making this an irrelevant point.

Secondly, Constantin‐Teodosiu suggests that the difference in steady‐state plasma carnitine concentration between the caffeine and placebo trials during the final 90 min of infusion was “minute” and would not measurably increase the muscle carnitine store. However, the author has demonstrated a fundamental misunderstanding of intravenous infusion experiments, where clearance of carnitine is measured as a rate over time and takes into account the entire volume of distribution for carnitine (which is extracellular water at around 20% of body mass), not the plasma volume. Again, it is not possible to determine a rate of clearance for carnitine from a single snapshot of plasma carnitine concentration and without factoring the rate of appearance of carnitine into the circulation. This misunderstanding is particularly puzzling given that these calculations have been laid out in prior publications demonstrating an analogous effect of insulin on plasma carnitine clearance and an associated increase in muscle carnitine content, work which Constantin‐Teodosiu is a named author on (Stephens et al., 2006, 2007). We note that Constantin‐Teodosiu has declared a conflict of interest around this work.

The final assertion of Constantin‐Teodosiu is that a caffeine mediated increase in muscle carnitine content would not be beneficial in type 2 diabetes, given that muscle carnitine content is elevated in type 2 diabetes and in healthy individuals following a high‐fat diet. Nowhere did we make such a claim. More disturbing is that closer inspection of the paper that Constantin‐Teodosiu quotes demonstrates no change in muscle carnitine content with high‐fat feeding, and no investigation of individuals with type 2 diabetes, making the critique spurious at best. We have previously demonstrated increased fat oxidation with an increase in muscle carnitine content in older insulin resistant individuals (Chee et al., 2021), and others have reported perturbed acylcarnitine metabolism in type 2 diabetes. We are surprised Constantin‐Teodosiu seems reluctant for further work delving into the role of carnitine metabolism in this population.

## ETHICS STATEMENT

None.
